# Short-term association between outdoor temperature and the hydration-marker copeptin: a pooled analysis in five cohorts

**DOI:** 10.1016/j.ebiom.2023.104750

**Published:** 2023-08-08

**Authors:** Simon Timpka, Olle Melander, Gunnar Engström, Sölve Elmståhl, Peter M. Nilsson, Lars Lind, Mats Pihlsgård, Sofia Enhörning

**Affiliations:** a Perinatal and Cardiovascular Epidemiology, Lund University Diabetes Centre, Department of Clinical Sciences in Malmö, Lund University, Malmö, Sweden; b Department of Obstetrics and Gynecology, Skåne University Hospital, Malmö, Sweden; c Department of Clinical Sciences in Malmö, Lund University, Malmö, Sweden; d Department of Internal Medicine, Skåne University Hospital, Malmö, Sweden; e Department of Clinical Sciences in Malmö, Division of Geriatric Medicine, Lund University, Malmö, Sweden; f Internal Medicine - Epidemiology, Department of Clinical Sciences Malmö, Lund University, Malmö, Sweden; g Department of Medical Sciences, Uppsala University, Uppsala, Sweden

**Keywords:** Cold environment, Heat, Temperature-related morbidity, Water intake, Vasopressin, Climate change, Copeptin

## Abstract

**Background:**

Whereas outdoor temperature is linked to both mortality and hydration status, the hormone vasopressin, measured through the surrogate copeptin, is a marker of cardiometabolic risk and hydration. We recently showed that copeptin has a seasonal pattern with higher plasma concentration in winter. Here, we aimed to investigate the association between outdoor temperature and copeptin.

**Methods:**

Copeptin was analysed in fasting plasma from five cohorts in Malmö, Sweden (n = 26,753, 49.7% men, age 18–86 years). We utilized a multivariable adjusted non-linear spline model with four knots to investigate the association between short-term temperature (24 h mean apparent) and log copeptin z-score.

**Findings:**

We found a distinct non-linear association between temperature and log copeptin z-score, with both moderately low and high temperatures linked to higher copeptin concentration (p < 0.0001). Between 0 °C and nadir at the 75th temperature percentile (corresponding to 14.3 °C), log copeptin decreased 0.13 z-scores (95% CI 0.096; 0.16), which also inversely corresponded to the increase in z-score log copeptin between the nadir and 21.3 °C.

**Interpretation:**

The J-shaped association between short-term temperature and copeptin resembles the J-shaped association between temperature and mortality. Whereas the untangling of temperature from other seasonal effects on hydration warrants further study, moderately increased water intake constitutes a feasible intervention to lower vasopressin and might mitigate adverse health effects of both moderately cold and hot outdoor temperatures.

**Funding:**

10.13039/501100004359Swedish Research Council, Å Wiberg, M Stephen, A Påhlsson, Crafoord and Swedish Heart-Lung Foundations, 10.13039/501100003748Swedish Society for Medical Research and 10.13039/501100007687Swedish Society of Medicine.

**Research in context**

**Evidence before this study**

Outdoor temperature is related to different measures of insufficient hydration in a J-shaped pattern, which mirrors the known J-shaped relationship between temperature and mortality, meaning that there is a higher risk of death and a less hydrated state at both moderately cold and hot temperatures.

Previous genetic and experimental data suggest a causal effect of the water-regulating hormone vasopressin on metabolic health. For example, human and animal experiments have shown beneficial metabolic effects from water-induced vasopressin reduction.

In a pooled analysis of Swedish population-based cohorts we recently found a distinct seasonal variation in the vasopressin surrogate marker copeptin, with elevated concentrations during the coldest months (mid-February to mid-March).

**Added value of this study**

The links between temperature and the water-regulating hormone vasopressin has not previously been studied in population-based cohorts. We present results suggesting a non-linear association between temperature and concentration of plasma copeptin, a surrogate marker of vasopressin, with higher concentrations at both moderately low and high outdoor temperatures.

**Implications of all the available evidence**

The J-shaped association between temperature and copeptin resembles the J-shaped association between temperature and mortality and mirrors the known association between temperature and other markers of hydration.

As vasopressin load in plasma can easily be lowered by increased water intake, our results suggest that increased water intake may be a novel, inexpensive, and widely available intervention to mitigate adverse health effects of both moderately cold and hot outdoor temperature.

## Introduction

The effects of temperature on human health have received increased attention during the last decade in parallel with the broader focus on climate change, which also affects freshwater resources.^1^ In total, almost five billion people already live in geographical areas with threatened water security.^2^ While insufficient hydration is usually considered to be a problem of heat, markers of underhydration have been linked to both relatively cold and very hot outdoor temperatures.^3^ This non-linear pattern resembles the relationship between temperature and mortality,^4^ meaning that there is a higher risk of death at both moderately cold and hot temperatures. While heat waves are detrimental to health, and especially so in the elderly,^5^ it has recently been reported that a substantially greater proportion of excess deaths is attributable to cold weather compared to heat in the United Kingdom.^6^

Vasopressin, also known as antidiuretic hormone (ADH), is commonly known as the key hormone in regulation of water balance by reducing renal water excretion. Vasopressin secretion decreases when serum osmolality decreases, is elevated in individuals with other indices of low water intake such as high urine osmolality, and is readily lowered by moderate water intake.^7^, ^8^, ^9^ There are also non-osmotic stimuli of vasopressin secretion such as stress.^10^ Vasopressin concentration is easily estimated through the surrogate marker copeptin,^11^ which thus is a marker of hydration and have enabled the identification of a link between the vasopressin system, cardiometabolic disease,^12^ and increased mortality rates.^13^, ^14^, ^15^ We have recently shown that copeptin has a distinct seasonal pattern in a temperate climate, with higher plasma concentration in winter of a magnitude corresponding to a relevant disease risk on the population level.^16^ However, the extent to which the outdoor temperature is associated with copeptin in a population-based setting is unknown.

To address this gap of knowledge, we studied the extent to which temperature is associated with copeptin plasma concentration, hypothesizing a higher plasma concentration of copeptin at both moderately low and high outdoor temperatures.

## Methods

### Study sample

We used data from five population based observational cohort studies conducted in Malmö, Sweden, during 1992–2018 (total n = 26,753, age 18–86 years, 49.7% men). The five cohorts are (I) the Malmö Diet and Cancer—Cardiovascular Cohort (MDC-CC, n = 5211, plasma samples collected in 1992–1994), (II) the Malmö Preventive Project (MPP, n = 5396, samples collected in 2002–2006), (III) the EpiHealth Malmö Cohort (n = 8217, samples collected in 2012–2017), (IV) the Malmö Offspring Study (MOS, n = 2243, samples collected in 2013–2018) and (V) the Swedish CArdioPulmonary BioImage Study Malmö Cohort (SCAPIS, n = 5686, samples collected in 2014–2018). All five cohorts have recruited participants fairly evenly distributed over the year, except for a much lower number of recruitments during the Swedish summer holiday season (Supplemental Table S1). The analysis set included all participants who had complete data on age, sex, copeptin, body mass index (BMI), blood sampling date and corresponding temperature data for that date. The sex of the study participants were based on the personal identity numbers in Swedish population registration. Detailed information about each cohort is published elsewhere.^16^

### Ethics

All studies were approved by the local ethical committees. Written informed consent was obtained from each participant. The pooled analyses were approved by the Swedish Ethical Review Authority (Dnr 2020-04422 and 2021-06180-02).

### Geographical location and climate

The city of Malmö (population 350,000) is located by the sea in southern Sweden. The altitude of the city is low (around 12 m) and urban Malmö is, in terms of surface area, not a very large city (approximately 75 km^2^). The climate is temperate with the mean temperature in the coldest months (January and February) of approximately 0 °C and in the warmest months (July and August) of approximately 20 °C. The relative air humidity reaches the lowest levels in June (around 70%) and the highest levels in December (around 85%).

### Analyses of copeptin plasma concentration

Copeptin was measured once at baseline in each of the study participants of the five cohorts.^17^ Brahms Copeptin proAVP KRYPTOR assay was used for analyses in the EpiHealth, SCAPIS and MOS cohorts, and Brahms CT-proAVP LIA assay was used in the MDC-CC and MPP cohorts. Fasting plasma samples were collected in EDTA plasma tubes and stored at −80 °C until analysis. Samples were drawn in all studies after an overnight fast, except in the EpiHealth cohort in which 6 h of fasting were required.

### Assessment of temperature variables

We collected data on temperatures and dew point temperatures in Malmö during the period of participants’ blood sampling in the city (1992–2018). Detailed data on temperature and dew point temperature from a meteorological station in Malmö (“Malmö A”) were obtained through freely available download via Swedish Meteorological and Hydrological Institute webpage (http://opendata-download-metobs.smhi.se/explore). To calculate mean apparent temperature 24 h before blood sampling, we used data points on temperature and dew point temperature every 3 h from 9 am the day before to 9 am on the day of sampling. To calculate apparent temperature, defined as a person’s perceived air temperature, we used the following formula: −2.653 + (0.994 × air temperature (°C)) + (0.0153 × dew point temperature2 (°C)).^18^ For brevity, we below use “temperature” synonymously with “apparent temperature” in reference to our data and results unless otherwise indicated.

### Statistical analyses

Copeptin was log-transformed due to skewed distribution which resulted in a variable more appropriate to use in regression analyses with continuous outcome.

Z-score was calculated based on sex and cohort as two different copeptin assays were used for analyses of plasma samples as described above. Results were thus presented as log z-score of copeptin standardized for cohort and sex. To investigate the relationship between temperature and copeptin concentration, we used regression models with mean temperature during the preceding 24 h as the exposure variable (24 h lag). To accommodate non-linearity, a spline model with knots at the 25th, 50th, 75th and 90th temperature percentiles was chosen based on literature review and reviewer feedback. Knots were primarily chosen to avoid small groups and because the 75th temperature percentile approximately reflects the temperature with lowest associated mortality rate, i.e. the minimum mortality temperature (MMT),^19^ which thus is likely to be physiologically relevant for overall homeostasis. However, recent data show that MMT in a temperate climate is closer to the 90th temperature percentile, which is why we also added a knot at the 90th temperature percentile into the model.^20^ To facilitate interpretation, we centered the curve at an approximate nadir at the 75th percentile (i.e. 14.3 °C). The multivariable models were adjusted for age, sex, body mass index (BMI), cohort, and day of the week. All 95% confidence intervals (CI) and tests were based on an asymptotic heteroscedasticity consistent variance estimator as we noted a minor deviance from standard regression model assumptions when QQ-plots and histograms were inspected visually. All statistical analyses were performed using SAS 9.4.

### Additional analyses

In addition to our main analysis, we also performed several complementary analyses.

We repeated the main analysis stratified by age (≤60 years and >60 years) and by sex, respectively, as the effect of temperature on health might differ by age and sex.

Moreover, as the data collection occurred during a period of roughly 25 years, it is possible that our results are affected by a time trend whichever the cause. We addressed this issue by expanding the model in the main analysis to include a cubic spline with 10 degrees of freedom of date. Furthermore, we adjusted our main model for seasonality by following the analysis in^16^ and updating the main model to include date through a sine and a cosine term with period one year.

To investigate the extent to which our results were driven by any particular cohort, we performed cohort specific analyses and conducted a leave-one-out analysis, in which one cohort at the time was dropped and the main analysis was repeated.

Finally, we ran the main analysis but used ambient instead of apparent temperature. Calculations and tests are further described in Supplemental Method description.

### Role of the funding sources

None of the funders of the study had any role in study design, collection, analysis or interpretation of data, or in the writing of the manuscript and they did not impact the decision to submit the paper for publication.

## Results

The pooled sample characteristics from the five cohorts are presented in Table 1 and separately by cohort in Table 2. Mean age was 59.5 years and 49.7% of participants were men.

**Table 1:** Pooled sample description of the five included cohorts.

N	26,753
Age, years	59.5 (10.5)
Men, n (%)	13,308 (49.7)
Body mass index,^a^ kg/m^2^	26.6 (23.6; 29.0)
Plasma copeptin,^a^ pmol/l	5.5 (3.6; 8.7)

Values are given as mean (standard deviation) if nothing else specified.

a Median (25th percentile; 75th percentile).

**Table 2:** Sample description per cohort.

MDC-CC	
N	5211
Age, years	57.6 (5.9)
Men, n (%)	2129 (40.9)
Body mass index,^a^ kg/m^2^	25.3 (23.0; 27.9)
Plasma copeptin,^a^ pmol/l	5.2 (3.2; 8.2)
MPP	
N	5396
Age, years	69.4 (6.2)
Men, n (%)	3768 (69.8)
Body mass index,^a^ kg/m^2^	26.7 (24.3; 29.4)
Plasma copeptin,^a^ pmol/l	7.2 (4.3; 11.9)
EpiHealth	
N	8217
Age, years	60.8 (8.4)
Men, n (%)	3656 (44.5)
Body mass index,^a^ kg/m^2^	25.8 (23.5; 28.7)
Plasma copeptin,^a^ pmol/l	5.0 (3.6; 7.8)
MOS	
N	2243
Age, years	39.8 (14.1)
Men, n (%)	1076 (48.0)
Body mass index,^a^ kg/m^2^	25.1 (22.7; 28.3)
Plasma copeptin,^a^ pmol/l	5.8 (3.8; 8.6)
SCAPIS	
N	5686
Age, years	57.5 (4.3)
Men, n (%)	2679 (47.1)
Body mass index,^a^ kg/m^2^	26.6 (24.1; 29.7)
Plasma copeptin,^a^ pmol/l	5.2 (3.6; 8.0)

Values are given as mean (standard deviation) if nothing else specified.

Abbreviations: MDC-CC, the Malmö Diet and Cancer—Cardiovascular Cohort; MPP, Malmö Preventive Project; EpiHealth, the EpiHealth Malmö Cohort; MOS, the Malmö Offspring Study; SCAPIS, the Swedish CArdioPulmonary BioImage Study Malmö Cohort.

a Median (25th percentile; 75th percentile).

**Fig. 1:**
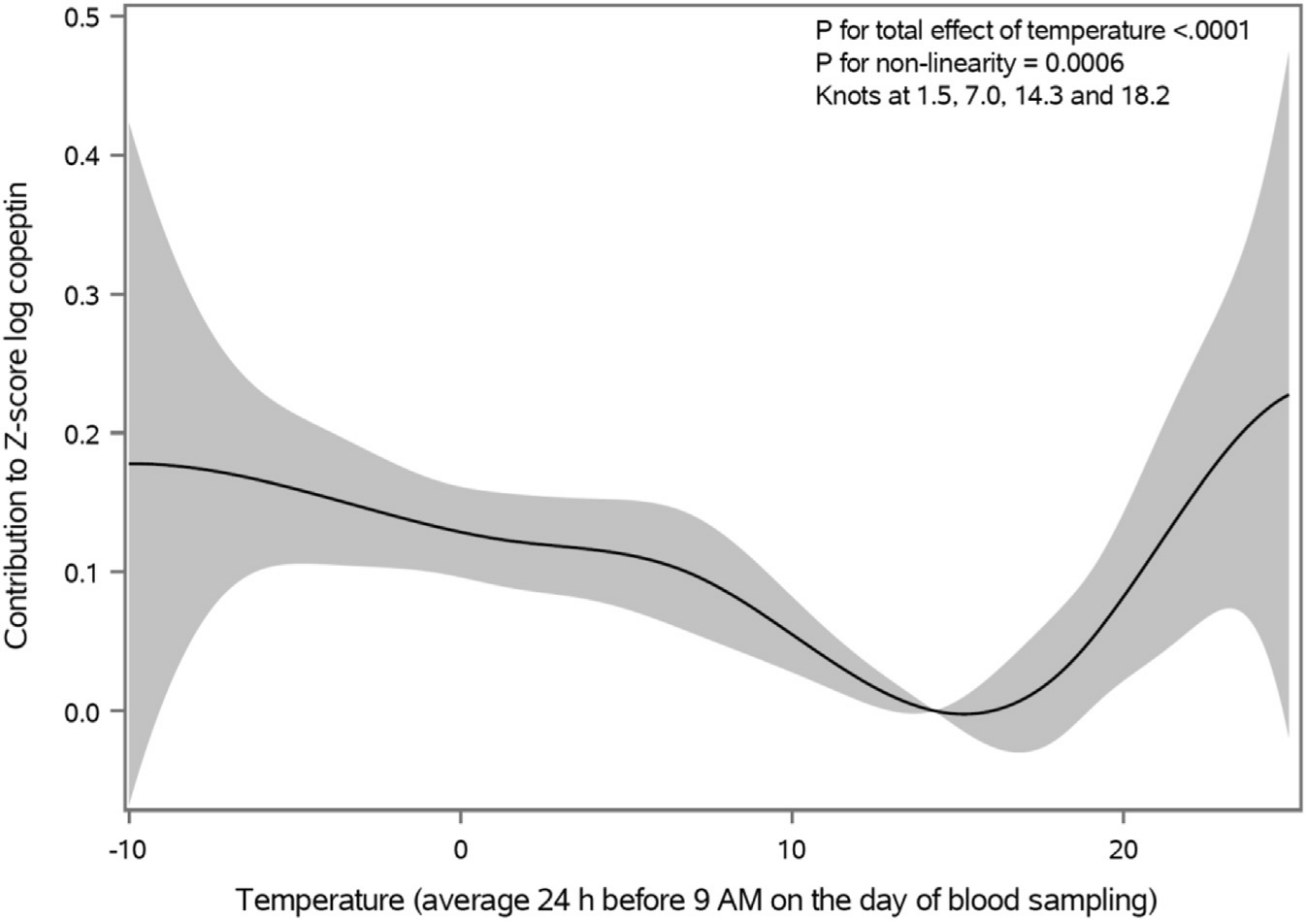
**Contribution of apparent temperature to plasma copeptin concentration.** The grey area demarcates the 95% confidence interval. Copeptin values are given as log copeptin z-score standardized for cohort and sex.

As shown in Table 3, the mean copeptin by temperature decile for days with at least one copeptin measurement peaked in the 1st temperature decile with a nadir at 8th and 9th temperature deciles. As shown in Fig. 1, the association between temperature and copeptin (p < 0.0001) was non-linear (p = 0.0006) and distinctly J-shaped. We observed a nadir at the third knot (the 75th temperature percentile, corresponding to a mean temperature of 14.3 °C), from which both decreased and increased temperature was associated with higher copeptin. Between 0 °C and the nadir, log copeptin concentration decreased 0.13 z-scores (95% CI 0.096; 0.16), which also inversely corresponded to the increase in z-score log copeptin between the nadir and 21.3 °C. If comparing the contribution of temperature to log copeptin concentration at the extreme ends of the temperature distribution (i.e. −10 °C and 25 °C) to the nadir (14.3 °C), the absolute difference was approximately 0.2 in z-score log copeptin.

**Table 3:** Temperature and copeptin by decile of temperature.

Temperature decile	Temperature, °C^a^			Copeptin	
	Mean (SD)	Minimum	Maximum	Mean log copeptin z-score (SD)^b^	Median copeptin (pmol/l)
1st	−4.1 (1.6)	−10.6	−2.1	0.08 (1.0)	5.8
2nd	−0.9 (0.6)	−2.0	0.1	0.05 (1.0)	5.6
3rd	1.2 (0.6)	0.1	2.2	0.05 (1.0)	5.5
4th	3.1 (0.5)	2.2	4.0	0.02 (1.0)	5.4
5th	5.1 (0.6)	4.0	6.2	0.04 (1.0)	5.5
6th	7.4 (0.8)	6.2	8.9	0.02 (1.0)	5.6
7th	10.1 (0.7)	8.9	11.4	−0.05 (1.0)	5.4
8th	12.7 (0.7)	11.4	13.9	−0.08 (1.0)	5.1
9th	15.2 (0.8)	13.9	16.6	−0.08 (1.0)	5.4
10th	18.8 (1.8)	16.6	25.6	−0.05 (1.0)	5.5

SD, standard deviation.

Only calendar days with at least one copeptin measurement during the study period 1992–2018 are included.

a Average 24 h apparent temperature.

b Given as log copeptin z-score standardized for cohort and sex.

As shown in Supplemental Figures S1 and S2, the decrease in copeptin with increasing temperature between 0 and 14.3 °C was greater in participants ≤60 years (vs. >60 years, 0.083 log copeptin z-score [95% CI 0.017; 0.15])) and women (vs. men, 0.078 log copeptin z-score [95% CI 0.013; 0.14]). The increase in copeptin with increasing temperature between 14.3 and 21.3 °C was greater in participants ≤60 years (vs. >60 years, 0.25 log copeptin z-score [95% CI 0.084; 0.42])) but a tendency towards a smaller increase was observed in women (vs. men, −0.14 log copeptin z-score [95% CI −0.31; 0.027]).

The contribution of temperature to copeptin remained similar after adjustment for long term time trend in the main model (Supplemental Figure S3). When season (sine and cosine terms) was introduced into the main model we found that the association between high temperature and elevated copeptin remained but that the non-linear relationship disappeared (Supplemental Figure S4). In the leave-one-out analysis we did not find any particular cohort to be the major driver of the association between temperature and elevated copeptin (Supplemental Figure S5), which was also evident when we analysed each of the cohorts separately (Supplemental Figure S6). Finally, the association remained significant but slightly attenuated when ambient (absolute) instead of apparent temperature was used in the analysis (Supplemental Figure S8).

## Discussion

Our main finding is that plasma copeptin concentration, a marker of hydration, is significantly and non-linearly associated with outdoor temperature and that this association is of a magnitude large enough to be relevant for human health on the population level. Given the plausible effect of high vasopressin on increased cardiometabolic disease risk and mortality, our results help to further link the observational evidence of higher cardiovascular morbidity and mortality at moderately cold temperatures to human physiology.

There is substantial evidence that the temperature with lowest associated mortality rate, the minimum mortality temperature (MMT), varies between locations and climates around the world.^19^ In general, a higher yearly mean temperature translates to a higher MMT,^20^ suggesting human adaptation to increasing mean temperature on the population-level. However, this adaptation seems incomplete, which is reflected by a lower MMT on the temperature-spectrum (i.e. a lower MMT percentile) with higher mean temperature.^20^ Pathophysiological explanations of how ambient temperature affects human health often focus on direct effects of temperature on body temperature and its physiological effects.^21^ While such changes might be induced by relatively extreme temperatures, they are less likely to occur at moderate temperatures in which community-dwelling adults can readily moderate their perceived temperature by clothing. Still, the most substantial effect of temperature on human health appears to be mediated be moderately cold temperatures.^19^

We have previously reported a winter peak of copeptin and hypothesized that it could be explained by a J-shaped association between temperature and copeptin concentration.^16^ The results of the current study supports this hypothesis. While we do not present data on other biochemical markers of hydration, previous evidence support our findings by suggesting a less hydrated state (underhydration) at both high and low temperatures, thereby providing a plausible explanation to the elevated vasopressin concentrations seen during winter.^3^ These investigators speculated that dehydration might strain the cardiovascular system, thereby mediating the temperature-related effects on mortality. We instead suggest that vasopressin, through its diverse hormonal effects, constitutes a mechanistic link between moderately cold temperatures, the associated underhydration, and increased rates of mortality and cardiovascular morbidity. While the water reabsorption in the renal collecting ducts are mediated mainly by the V2 receptor,^22^ the metabolic and cardiovascular effects of vasopressin are also mediated via V1a and V1b receptors.^23^ As we have previously summarized,^16^ these include, but are not limited to, gluconeogenesis, anti-lipolysis, and platelet aggregation. In contrast to the effects of heat, cold appears to have effects on mortality over longer periods.^19^ Given the hormonal effects of vasopressin on metabolism and cardiovascular health and the longer time interval of effect this entails, our results should expand the current framework to understand the mechanistic effects of temperature on cardiovascular morbidity and mortality.^21^ As vasopressin is readily lowered through moderate water intake,^9^ our observations suggest that increased water intake could partly mitigate the detrimental health effects caused by moderately cold temperatures.

When season—a proxy for all variables varying by season including other meteorological variables, infectious diseases, changes in diet and other behavioral patterns, as well as temperatures of other lags than the investigated—was introduced into the main model, we found that the association between high temperature and elevated copeptin remained but that the non-linear relationship disappeared (Supplemental Figure S4). This meant that only higher temperature contributed to elevated copeptin concentration, whereas the inverse cold effect disappeared. A non-j-shaped increase in copeptin concentration with increased temperature resembles the conventional understanding of the vasopressin system and is congruent to past findings from smaller studies showing higher vasopressin concentration during warm compared to cold season.^24^^,^^25^ Our current results may thus facilitate the merging of our previous finding of a seasonal effect on copeptin concentration with past findings of higher vasopressin during warm season. Speculatively, the contribution of low outdoor temperature to elevated copeptin, which disappears after adjustment for season, may be linked to more long-term mechanisms (behavioral or meteorological).

During hot days, an increase in vasopressin secretion might be part of an adequate physiological response to conserve body water. During moderately cold days, the higher vasopressin concentration might instead be an indicator of a less hydrated state, or at least insufficient water intake to adequately suppress vasopressin secretion. One potential explanation to negative water balance and increased vasopressin concentration during colder days is cold-induced diuresis. The underlying mechanism(s) behind cold-induced diuresis is not completely understood. In a previous human experiment, it was shown that short-term cold exposure (1 h) attenuated thirst and lowered vasopressin concentration despite a condition of underhydration and elevated plasma osmolality.^26^ Other experimental evidence suggest that low outdoor temperature decreases thirst.^27^ Whereas the underlying mechanism may be central volume expansion mimicking volume expansion, which in turn affects vasopressin secretion and thirst,^22^ oral cooling also increases the thirst quenching effect of fluids.^28^ An experiment in rats suggests that cold exposure decreases renal VP 2 receptor mRNA expression and results in increased urine output, while vasopressin concentration remain unaltered over 5 weeks (despite increased urine output).^29^ The downregulation of V2R in the kidney would be expected to result in a negative water balance (and thus increased vasopressin concentration), especially in combination with decreased thirst.

Another possibility behind temperature-related vasopressin secretion is non-osmotic stimuli such as stress.^10^ It is previously suggested that vasopressin regulates stress-induced catecholamine release via V1b receptors,^30^ that sympathetic nerve activity is higher during the cold season^31^ and that pressor response to cold stress may be implicated in health risks.^32^

Additional analyses stratified on age suggested that those older than age 60 years had an attenuated pattern of temperature-copeptin association compared to those age 60 years or younger. Older adults are at higher risk of temperature-related mortality^33^ and our results might help facilitate future studies to further understand this susceptibility. As we also found some evidence of a different temperature-copeptin association by sex, the vasopressin-system warrants further consideration in studies disentangling sex-specific effects of temperature on health and mortality risk.^33^

As humidity is relevant for the physiological response to ambient temperature,^34^ we used apparent temperature, which incorporates both temperature and dew point temperature, as our main temperature exposure. When using temperature instead of apparent temperature in our analysis, our main results remained similar albeit somewhat attenuated.

The 0.2 difference in z-score log copeptin across the temperature spectrum needs to be put in context to be interpretable. We have previously reported that a similar difference in standardized log copeptin was associated with a four percent higher risk of diabetes and a two percent higher risk of incident coronary artery disease^16^ and that 1 SD difference in log copeptin was associated with a 31% increased risk of mortality in the MPP Cohort,^13^ meaning that 0.2 SD would translate to approximately six percent increased risk of mortality.

Our study also has certain limitations that should be considered when interpreting the results. Firstly, we defined spline knots based on temperature percentiles. However, this methodology does not allow for exact estimation of the temperature nadir of copeptin concentration. Still, the nadir at the 75th temperature percentile also approximately reflects the average MMT,^19^ indicating that the knot demarcate temperature strata relevant for human health. Secondly, blood sampling in the study cohorts was typically performed following an overnight fast. However, previous evidence indicate that copeptin concentration is not particularly dependent on the timing of sampling,^35^ suggesting that the copeptin analyses of our morning samples reasonably reflects vasopressin load throughout the day. Thirdly, as we focused on short-term temperature effects on copeptin in this study, it remains unknown the extent to which temperature during longer lags is associated with plasma copeptin concentration.

In order to modify public health recommendations of water intake by temperature, the observational data we here present warrants additional support by evidence from intervention studies. In an ongoing randomized clinical trial (clinicaltrials.gov: NCT03422848), we are currently investigating the cardiometabolic effects of lowering vasopressin concentration through moderately increased water intake in participants with high copeptin. While coaching to increase water intake among adults with reduced kidney function (eGFR 30–60 mL/min/1.73 m^2^) resulted in no reduction in kidney function decline,^36^ future studies should investigate the extent to which other high-risk groups, e.g. those with clinical cardiovascular disease or diabetes, might benefit from targeted interventions of lowering vasopressin concentration to increase cardiometabolic health. Our results also highlight that access to clean and readily available drinking water is a public health issue that is not limited to areas with hot weather or unsanitary sources of freshwater.

### Conclusion

The J-shaped association between short-term temperature and copeptin resembles the J-shaped association between temperature and mortality. Whereas the untangling of temperature from other seasonal effects on hydration warrants further study, moderately increased water intake constitutes a feasible intervention to lower vasopressin and might mitigate adverse health effects of both moderately cold and hot outdoor temperatures.

## Contributors

Timpka, Pihlsgård and Enhörning designed the study. Pihlsgård conducted the statistical analyses and Timpka and Enhörning verified the data. Timpka and Enhörning funded the research and wrote the first draft of the manuscript. Melander, Engström, Lind, Elmståhl and Nilsson were involved in the data collection and running of the different cohorts included in the manuscript and contributed to the writing of the manuscript. All authors critically reviewed the final draft of the manuscript. Timpka and Enhörning were responsible for the decision to submit the manuscript.

## Data sharing statement

Data described in the manuscript and the analytic code used for the analyses are available upon reasonable request to each of the cohorts’ principal investigator or to the authors, respectively.

## Declaration of interests

Dr Melander has received a research grant and consultancy fee from Danone Research. Dr Enhörning has participated and participates in ongoing research trials partly funded by Danone Research. The authors report no other competing interests in this work.

## Appendix A Supplementary data

Supplemental Method
