# Supplementary material for: Short-term association between outdoor temperature and the hydration-marker copeptin: a pooled analysis in five cohorts
Source: eBioMedicine. 2023 Aug 8;95:104750. doi: 10.1016/j.ebiom.2023.104750 (PMC10432996; doi:10.1016/j.ebiom.2023.104750)
Supplement: Supplemental Method [file mmc1.pdf]

Supplement to "Short-term association between outdoor temperature and the hydration-marker copeptin: a pooled analysis in five cohorts" by Timpka *et al.*

Table of contents

Z-score for logarithm of copeptin 2
Basic regression analysis setup 2
Main analysis 2
Pooling of data and regression outcomes from different cohorts 3
Supplemental figures and tables 4
Supplemental references 12

Z-score for logarithm of copeptin

Let c_i be the measurement of copeptin for the ith patient and $l_i = \log(c_i)$. We construct strata (stratum 1,..., stratum 10) by combining cohort and sex. Patient i belongs to stratum $j = j(i)$. We let μ_j and σ_j denote the mean and standard deviation of logarithm of copeptin corresponding to stratum j. We compute the z-score, z_i, for each patient according to

$$z_i = (l_i - \mu_{j(i)})/\sigma_{j(i)}. \tag{1}$$

Basic regression analysis setup

We observe a vector $y = X\beta + \varepsilon$ for a known $n \times p$ matrix $X = (X_1 \dots X_p)$ of full rank. ε is an error vector which we assume to have independent components with mean zero and finite (but not necessarily equal) variances. The least-squares estimator of β is

$$\widehat{\beta} = (X'X)^{-1}X'y \tag{2}$$

(X' denotes the transpose of the matrix X). It is well known that $\widehat{\beta}$ is unbiased. We use the heteroscedasticity consistent covariance estimator of $\widehat{\beta}$ introduced in [1], which is given by

$$\widehat{\mathbb{C}ov\widehat{\beta}} = (X'X)^{-1}(X'DX)(X'X)^{-1}, \tag{3}$$

where D is the diagonal matrix with squares of regression residuals as non-zero elements. It follows by the discussion in [2], pp. 55–58, that random sampling and large n leads to

$$\widehat{\beta} \mathrel{\dot\sim} N(\beta, \widehat{\mathbb{C}ov\widehat{\beta}}), \tag{4}$$

where "$\dot\sim$" should be read "is approximately distributed as" and carries no mathematically rigorous meaning.

Main analysis

Let the spline knots be denoted k_1, k_2, k_3, k_4. We build a cubic polynomial spline basis of temperature by letting X_{i1} be the temperature corresponding to patient i, $X_{i2} = X_{i1}^2$, $X_{i3} = X_{i1}^3$, $X_{i4} = \max(X_{i1} - k_1, 0)^3$, $X_{i5} = \max(X_{i1} - k_2, 0)^3$, $X_{i6} = \max(X_{i1} - k_3, 0)^3$, $X_{i7} = \max(X_{i1} - k_4, 0)^3$ and $X_{i8} = 1$.[1] The remaining columns (X_9–X_{19}) correspond to body mass index, age, cohort, sex and day of the week of blood sampling (we ensure full rank of X by choosing reference levels for cohort, sex and day of the week of blood sampling). We perform two hypothesis tests. To this end, let L_i denote the row vector of length 19 with ith element equal to one and zero otherwise and

$$C_1 = \begin{bmatrix} L_1 \\ L_2 \\ L_3 \\ L_4 \\ L_5 \\ L_6 \\ L_7 \end{bmatrix}, \; C_2 = \begin{bmatrix} L_2 \\ L_3 \\ L_4 \\ L_5 \\ L_6 \\ L_7 \end{bmatrix}.$$

[1]It is always possible to replace the polynomial spline basis by an equivalent Bézier spline basis. The latter has potentially better numerical properties. However, we ran all models using both bases and found no indication of significantly better performance for the latter. As the results related to the former are easier to interpret we prefer this.

The first one ("Total effect of temperature") corresponds to the null hypothesis $H_0 : C_1\beta = 0$ and alternative hypothesis $H_1 : C_1\beta \neq 0$. The second one ("Non-linear effect of temperature") corresponds to $H_0 : C_2\beta = 0$ and $H_1 : C_2\beta \neq 0$. We compute the Wald statistics corresponding to C_1 and C_2 according to

$$W_1 = (C_1\widehat{\beta})'(C_1\widehat{\mathbb{C}ov\widehat{\beta}}C_1')^{-1}(C_1\widehat{\beta}), \tag{5a}$$

$$W_2 = (C_2\widehat{\beta})'(C_2\widehat{\mathbb{C}ov\widehat{\beta}}C_2')^{-1}(C_2\widehat{\beta}). \tag{5b}$$

Under the null hypotheses stated above, it holds (see [2], p. 58) that $W_1 \dot{\sim} \chi^2(7)$ and $W_2 \dot{\sim} \chi^2(6)$, respectively, and it is straightforward to compute approximate p-values for the hypothesis tests.

Pooling of data and regression outcomes from different cohorts

Two pooled analyses are performed.

1. Data from all cohorts are pooled and the analysis is conducted subsequently (main analysis).
2. Each cohort is analyzed separately (without adjustment for cohort!) and the resulting outcomes are combined.

Let us briefly describe the second approach. For convenience, we use the notation $Co_1, \ldots, Co_5$ for the cohorts. For each i, $i = 1, \ldots, 5$, we let y_i, X_i, β_i etc., be the equivalents of y, X, β,..., corresponding to Co_i. Note that X_i has only 15 columns. We let the first eight columns in X_i correspond to the same explanatory variables as X_1–X_8 in the main analysis, $\beta_{\text{tot}} = [\beta_1' \ \beta_2' \ \beta_3' \ \beta_4' \ \beta_5']'$ and $\widehat{\beta_{\text{tot}}} = [\widehat{\beta_1}' \ \widehat{\beta_2}' \ \widehat{\beta_3}' \ \widehat{\beta_4}' \ \widehat{\beta_5}']'$. Clearly, the covariance of $\widehat{\beta_{\text{tot}}}$ has a block diagonal structure according to

$$\widehat{\mathbb{C}ov\widehat{\beta_{\text{tot}}}} = \begin{bmatrix} \widehat{\mathbb{C}ov\widehat{\beta_1}} & 0 & 0 & 0 & 0 \\ 0 & \widehat{\mathbb{C}ov\widehat{\beta_2}} & 0 & 0 & 0 \\ 0 & 0 & \widehat{\mathbb{C}ov\widehat{\beta_3}} & 0 & 0 \\ 0 & 0 & 0 & \widehat{\mathbb{C}ov\widehat{\beta_4}} & 0 \\ 0 & 0 & 0 & 0 & \widehat{\mathbb{C}ov\widehat{\beta_5}} \end{bmatrix}.$$

Let L_k denote the row vector of length 15 with kth element equal to one and zero otherwise. Take w_i to be a weight corresponding to Co_i and let $c_i = w_i / \sum w_i$. For each set of weights we obtain a pooled estimate $\widehat{\beta_{\text{pool}}}$ of β by taking

$$\widehat{\beta_{\text{pool}}} = \begin{bmatrix} c_1L_1 & c_2L_1 & c_3L_1 & c_4L_1 & c_5L_1 \\ c_1L_2 & c_2L_2 & c_3L_2 & c_4L_2 & c_5L_2 \\ \vdots & \vdots & \vdots & \vdots & \vdots \\ c_1L_{15} & c_2L_{15} & c_3L_{15} & c_4L_{15} & c_5L_{15} \end{bmatrix} \widehat{\beta_{\text{tot}}}. \tag{6}$$

If we denote the matrix in (6) by W, we get that

$$\widehat{\mathbb{C}ov\widehat{\beta_{\text{pool}}}} = W\widehat{\mathbb{C}ov\widehat{\beta_{\text{tot}}}}W'. \tag{7}$$

Based on (6) and (7), we can, e.g., run hypothesis tests similar to "Total effect of temperature" and "Non-linear effect of temperature". By taking $w_i = n_i$, where n_i is the number of patients in Co_i, we expect to obtain results similar to what we saw in the main analysis.

Supplemental figures and tables

Abbreviations used throughout the section: EpiHealth, the EpiHealth Malmö Cohort; MDC-CC, the Malmö Diet and Cancer – Cardiovascular Cohort; MOS, the Malmö Offspring Study; MPP, Malmö Preventive Project; SCAPIS, the Swedish CArdioPulmonary BioImage Study Malmö Cohort. In all figures curves represent point estimates and shaded areas demarcate 95% confidence intervals.

Figure 1 and Figure 2 illustrate the contribution of outdoor temperature to z-score of logarithm of plasma copeptin concentration assessed through stratified analyses in individuals ≤ 60 years of age (Figure 1a), in individuals > 60 years of age (Figure 1b), in women (Figure 2a) and in men (Figure 2b), respectively. Let m_{1a}, m_{1b}, m_{2a} and m_{2b} be the corresponding mean differences between 0 and 14·3 degrees C and $\Delta_1 = m_{1a} - m_{1b}$ and $\Delta_2 = m_{2a} - m_{2b}$. Estimates and 95% confidence intervals for these parameters are, respectively,

$$\widehat{m_{1a}} = 0{\cdot}16; \quad I_{m_{1a}} = (0{\cdot}11, 0{\cdot}21), \quad \widehat{m_{1b}} = 0{\cdot}077; \quad I_{m_{1b}} = (0{\cdot}029, 0{\cdot}12), \quad (8a)$$

$$\widehat{m_{2a}} = 0{\cdot}16; \quad I_{m_{2a}} = (0{\cdot}12, 0{\cdot}21), \quad \widehat{m_{2b}} = 0{\cdot}087; \quad I_{m_{2b}} = (0{\cdot}040, 0{\cdot}13), \quad (8b)$$

$$\widehat{\Delta_1} = 0{\cdot}083; \quad I_{\Delta_1} = (0{\cdot}017, 0{\cdot}15), \quad \widehat{\Delta_2} = 0{\cdot}078; \quad I_{\Delta_2} = (0{\cdot}013, 0{\cdot}14). \quad (8c)$$

If we replace 0 by 21·3 in the definitions above, we get

$$\widehat{m_{1a}} = 0{\cdot}22; \quad I_{m_{1a}} = (0{\cdot}11, 0{\cdot}33), \quad \widehat{m_{1b}} = \text{-}0{\cdot}028; \quad I_{m_{1b}} = (\text{-}0{\cdot}15, 0{\cdot}096), \quad (9a)$$

$$\widehat{m_{2a}} = 0{\cdot}055; \quad I_{m_{2a}} = (\text{-}0{\cdot}062, 0{\cdot}17), \quad \widehat{m_{2b}} = 0{\cdot}19; \quad I_{m_{2b}} = (0{\cdot}075, 0{\cdot}31), \quad (9b)$$

$$\widehat{\Delta_1} = 0{\cdot}25; \quad I_{\Delta_1} = (0{\cdot}084, 0{\cdot}42), \quad \widehat{\Delta_2} = \text{-}0{\cdot}14; \quad I_{\Delta_2} = (\text{-}0{\cdot}31, 0{\cdot}027). \quad (9c)$$

Note that the information provided in (8a), (8b), (9a) and (9b) is available in Figure 1 and Figure 2, whereas (8c) and (9c) require calculations. The outcome of the analysis with adjustment for time trend is displayed in Figure 3. The seasonality adjusted results are shown in Figure 4. The leave-one-out analysis is illustrated in Figure 5. Per cohort analyses are shown in Figure 6. A comparison between the outcome of the main analysis (with regression using pooled data) and the outcome of pooling of regression parameters (corresponding to different cohorts) is found in Figure 7. The results coming from the main model with absolute temperature instead of apparent temperature are shown in Figure 8. Between 0 degrees C and the nadir at 15·5 degrees C, mean z-score of logarithm of copeptin concentration decreased 0·14 (95% confidence interval (0·10, 0·18)), which corresponds to the increase in mean z-score of logarithm of copeptin between the nadir and 20·4 degrees C. Figure 9 contains a flowchart showing the original sample size and final study sample in each of the cohorts. Finally, in Table 1 we display the number of copeptin measurements per month per cohort.

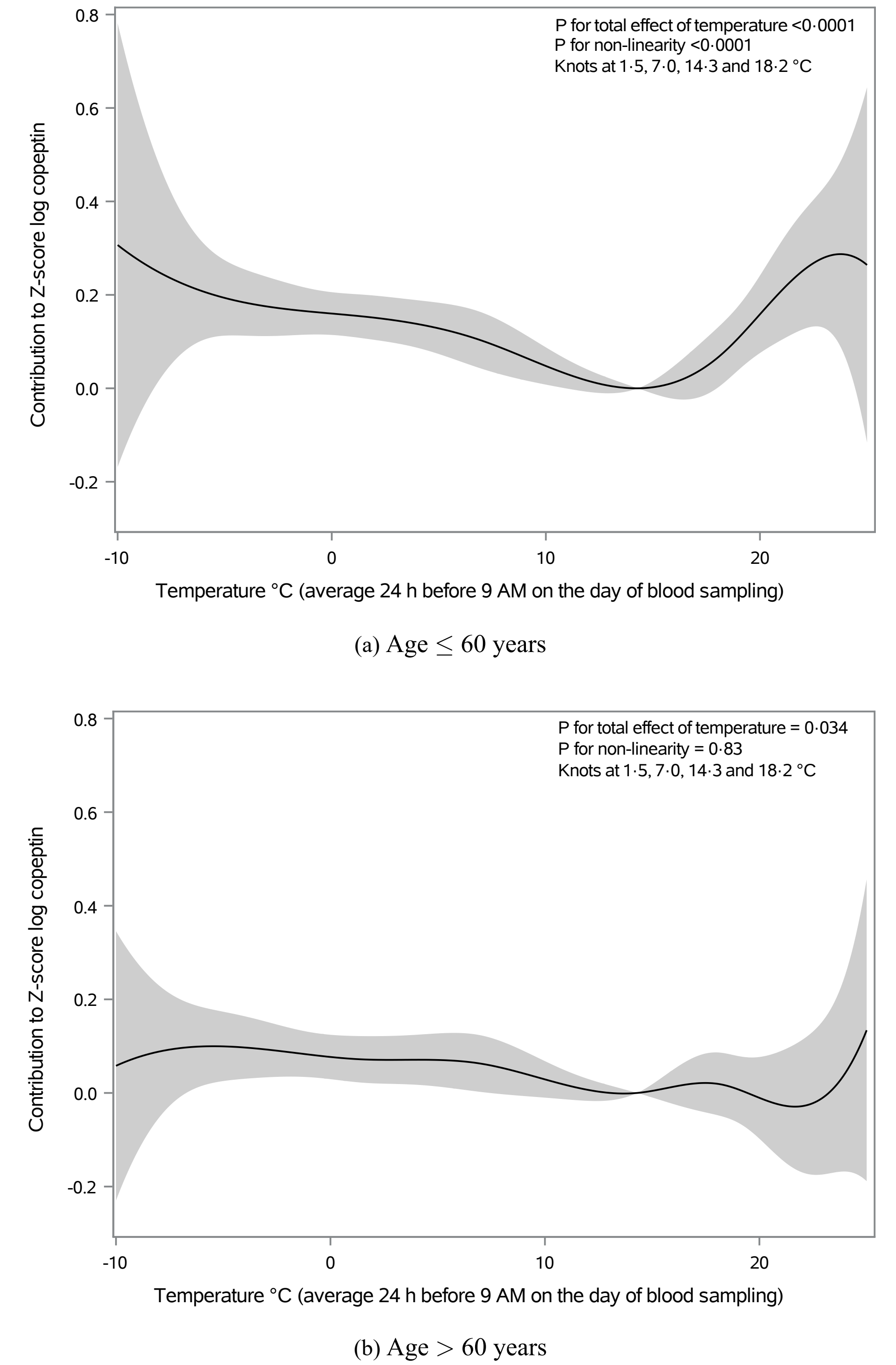

(a) Age $\leq$ 60 years

(b) Age $>$ 60 years

Supplemental Figure 1: Association between temperature and copeptin stratified by age

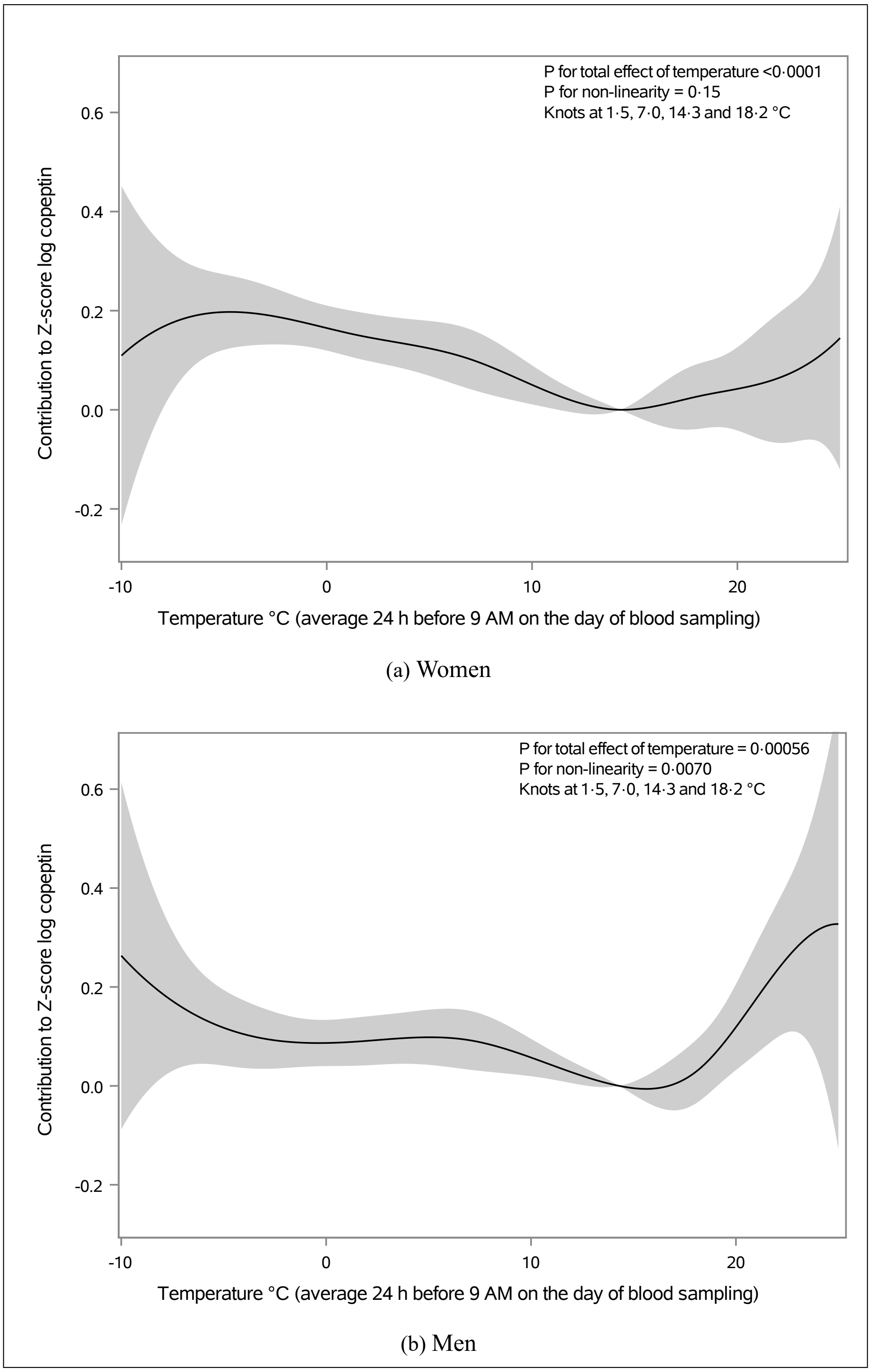

Supplemental Figure 2: Association between temperature and copeptin stratified by sex

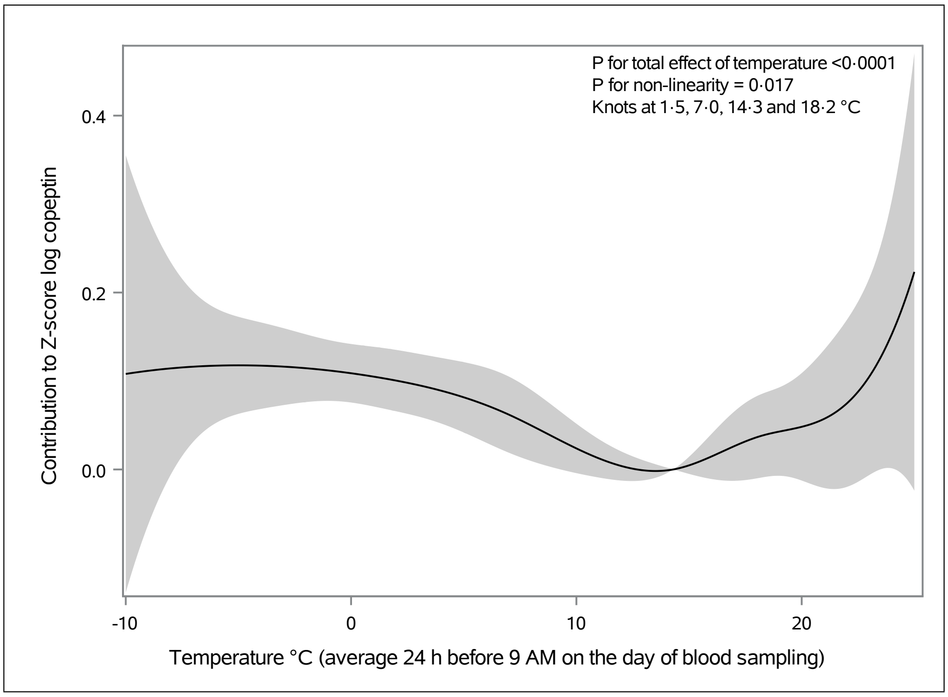

Supplemental Figure 3: Association between temperature and copeptin with adjustment for time trend

	EpiHealth	MDC-CC	MOS	MPP	SCAPIS
**January**	658	405	191	369	552
**February**	710	462	236	369	586
**March**	810	712	248	624	559
**April**	782	744	198	395	459
**May**	816	585	185	586	503
**June**	632	397	209	150	255
**July**	409	0	89	0	0
**August**	416	258	154	510	596
**September**	783	420	150	1064	649
**October**	885	483	210	449	612
**November**	757	365	209	659	579
**December**	559	380	164	221	336

Supplemental Table 1: Copeptin measurements per month per cohort

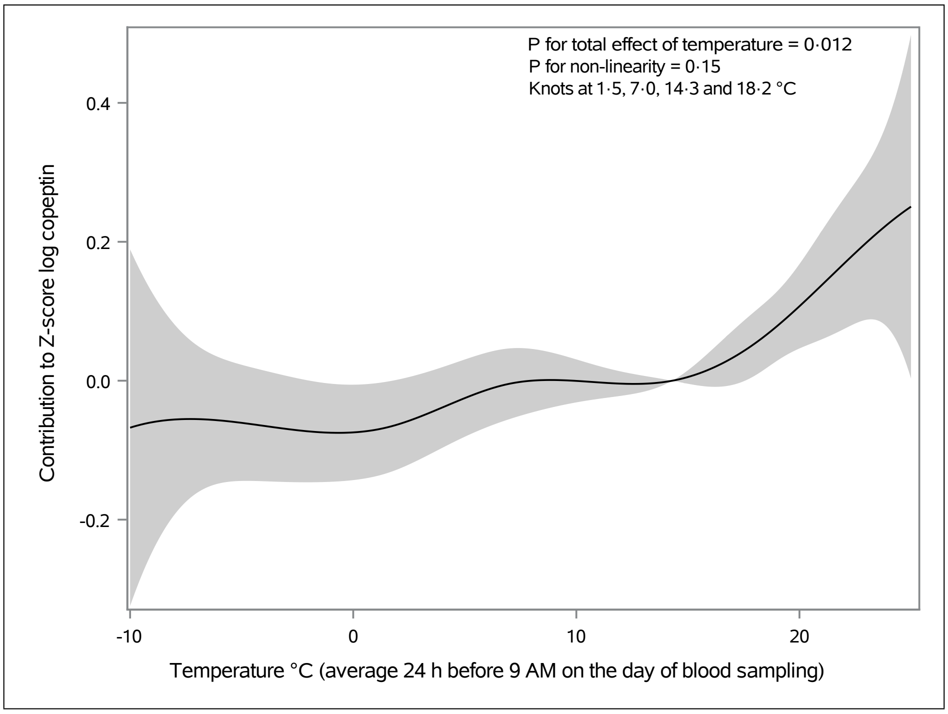

Supplemental Figure 4: Association between temperature and copeptin with adjustment for seasonality

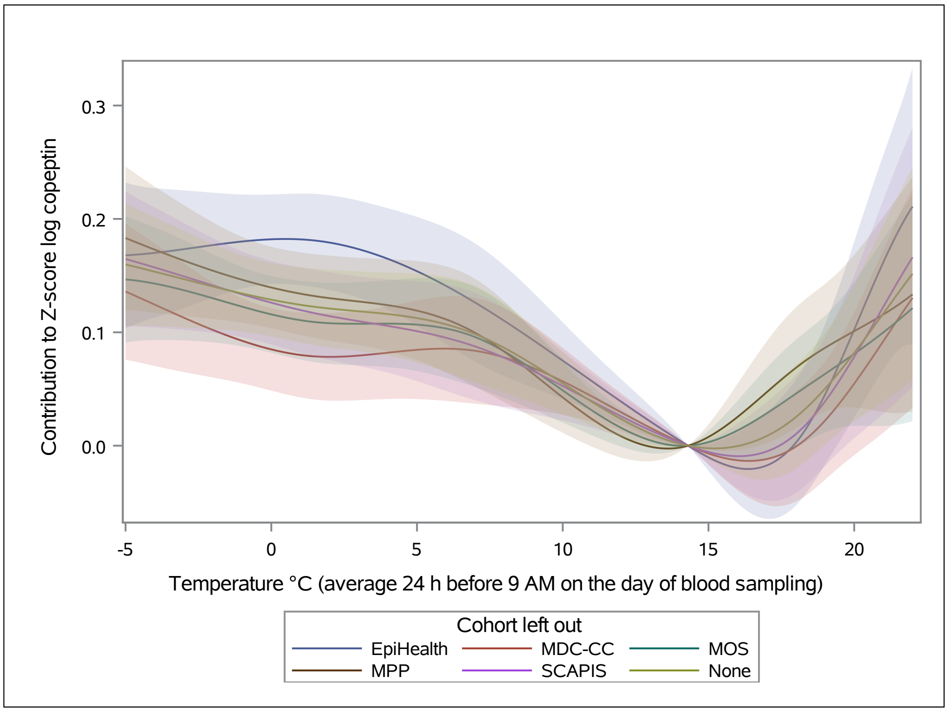

Supplemental Figure 5: Association between temperature and copeptin in leave-one-out analyses by cohort

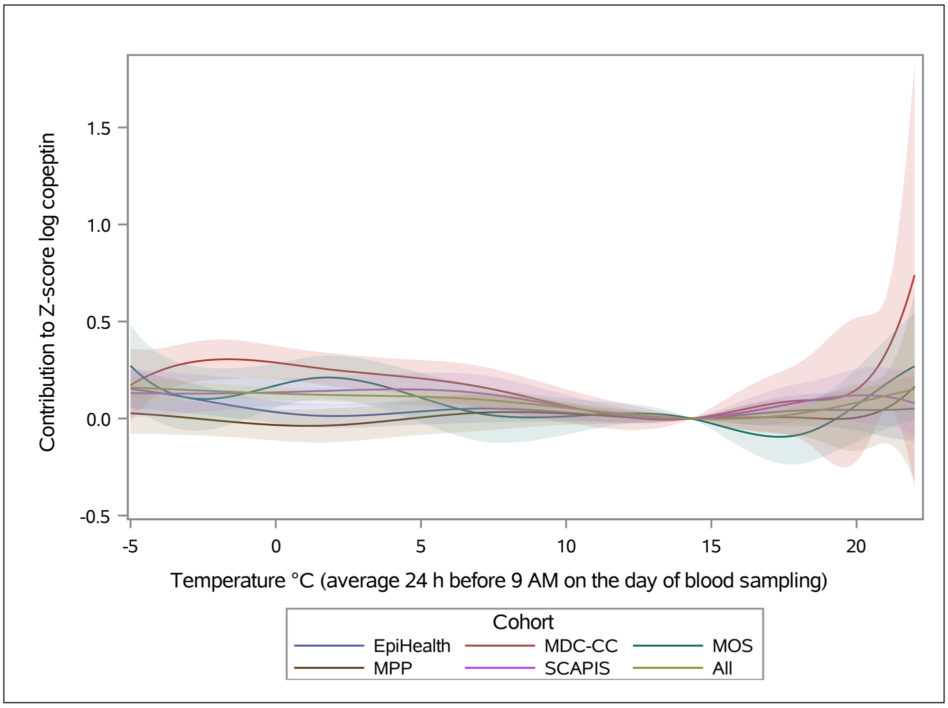

Supplemental Figure 6: Association between temperature and copeptin by cohort

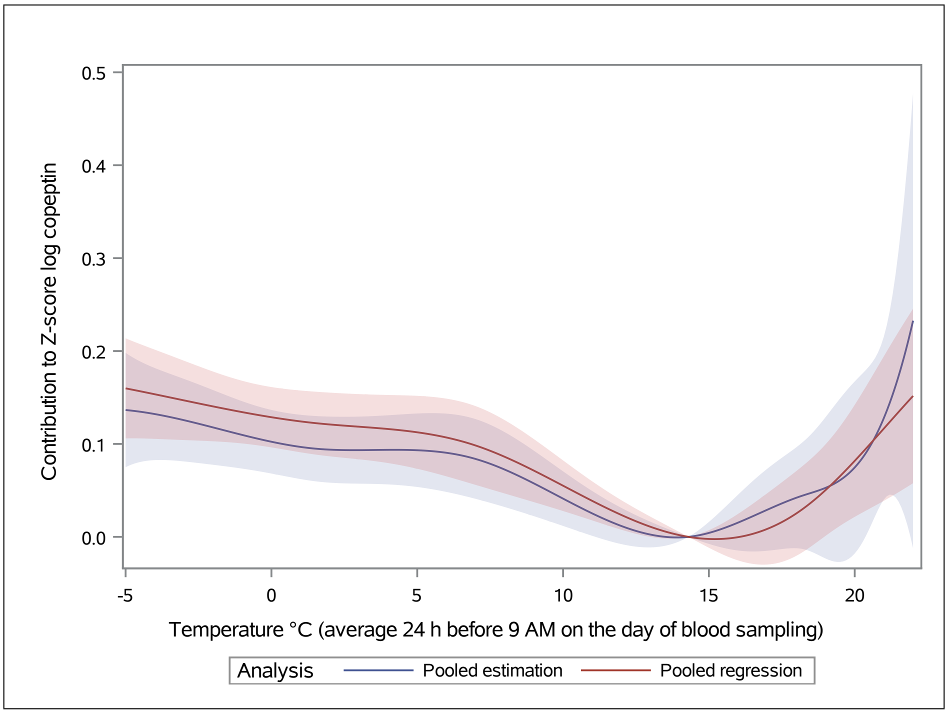

Supplemental Figure 7: Pooled estimates (weighted by cohort sizes) compared to results from regression based on pooled data

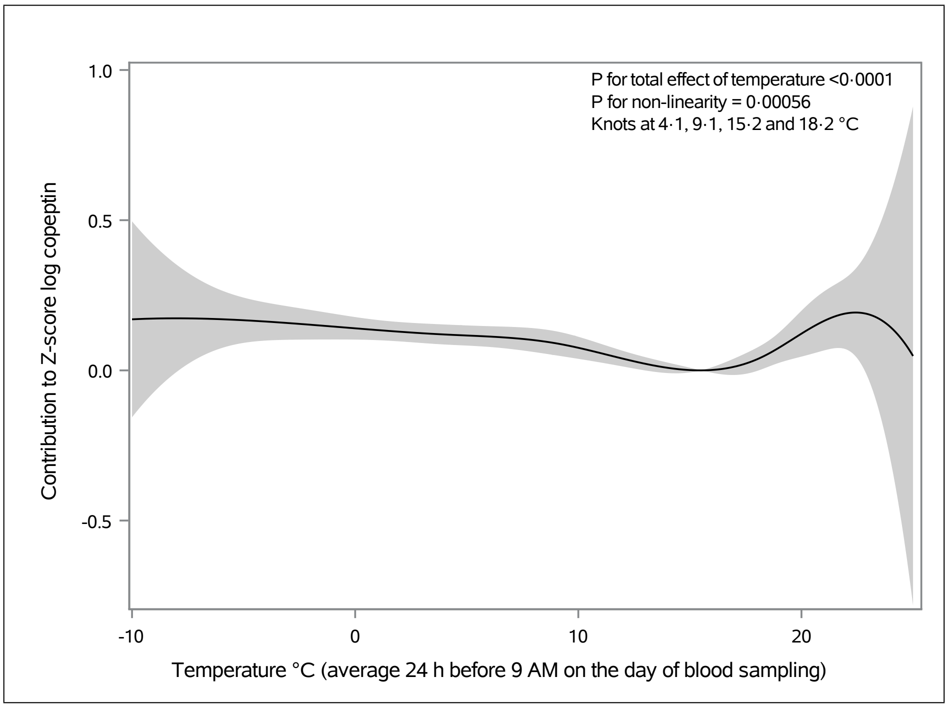

Supplemental Figure 8: Main analysis of contribution of temperature to copeptin concentration repeated with absolute instead of apparent temperature as exposure

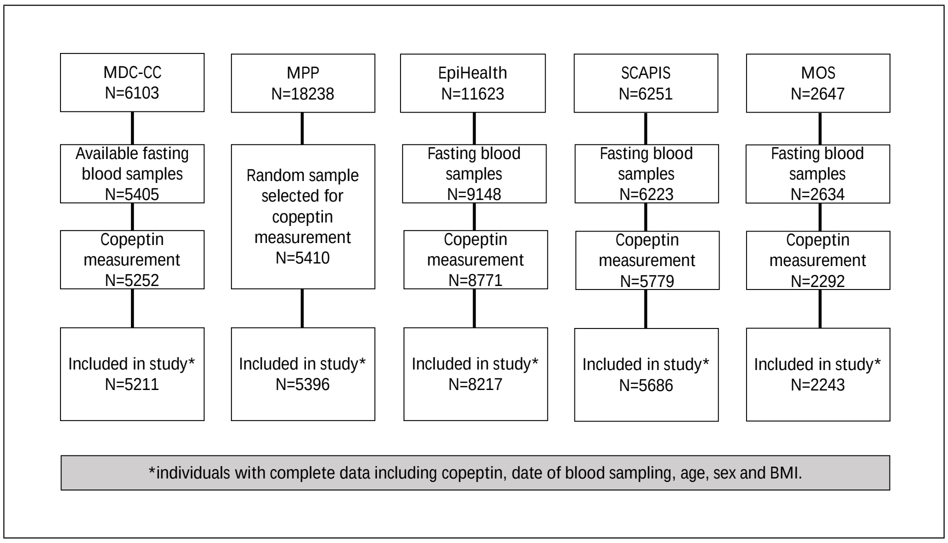

Supplemental Figure 9: Flowchart of original sample size and final study sample in each of the cohorts

Supplemental references

[1] White H. A heteroskedasticity-consistent covariance matrix estimator and a direct test for heteroskedasticity. Econometrica 1980; 48: 817–38.

[2] Fahrmeir L, Tutz G. Multivariate statistical modelling based on generalized linear models. 2nd ed. New York: Springer, 2001.

[3] Enhörning S, Melander O, Engström G, Elmståhl S, Lind L, Nilsson PM, Pihlsgård M, Timpka S. Seasonal variation of vasopressin and its relevance for the winter peak of cardiometabolic disease: A pooled analysis of five cohorts. J Intern Med 2022; 292: 365-76.
